# Anterolateral Ligament and Kaplan Fiber Injury Both Occur Frequently in Acute Anterior Cruciate Ligament-Injured Knees

**DOI:** 10.3390/jcm13040946

**Published:** 2024-02-07

**Authors:** Renata Vidal Leao, Paulo Victor Partezani Helito, Adnan Saithna, Marcos Felippe de Paula Correa, Camilo Partezani Helito

**Affiliations:** 1 Institute of Radiology, Hospital Sírio-Libanês, R. Ovidio Pires de Campos, São Paulo 05403-911, Brazil; marcos.fcorrea@hsl.org.br; 2 Department of Radiology, Aspetar Qatar Orthopaedic and Sports Medicine Hospital, Doha P.O. Box 29222, Qatar; 3 AZBSC Orthopedics, 7649 E Pinnacle Peak Rd, Scottsdale, AZ 85255, USA; adnan.saithna@gmail.com; 4 Orthopaedics Department, Arizona State University, Tempe, AZ 85287, USA; 5 Institute of Orthopaedics and Traumatology, Faculty of Medicine, University of São Paulo, São Paulo 05508-220, Brazil; camilo.helito@hc.fm.usp.br

**Keywords:** anterior cruciate ligament tears, anterolateral ligament, Kaplan fibers

## Abstract

Background and objectives: The association of ALL and KF injuries in anterior cruciate ligament (ACL)-deficient knees remain topics of conflicting research despite improved magnetic resonance imaging (MRI). We aimed to evaluate the rate of the anterolateral ligament (ALL) and Kaplan fibers (KF) injuries in adults with acute ACL injuries using MRI. Methods: We retrospectively reviewed 64 patients with clinical and MRI diagnoses of acute ACL tears. Two radiologists analyzed and categorized the status of the ALL and KF in all patients as intact, partially injured, or completely injured. Interobserver agreement was assessed. Injuries to the collateral ligaments, ITB and posterior cruciate ligament (PCL) were also evaluated. Results: The mean age of the patients was 33 years. ALL injuries were observed in 46 (71%) patients, among whom 33 (71%) had partial and 13 (28%) had complete injuries. KF injuries were identified in 32 (50%) patients, with 28 (87.5%) of them having partial and 4 (12.5%) having complete injuries. Combined injuries of both ALL and KF were found in 25 (32.4%) patients (*p*-value of 0.266). The agreement between the examiners ranged from moderate to substantial (Kappa between 0.55 and 0.75), with the highest agreement observed in cases of KF injuries (Kappa = 0.75). Conclusions: ALL and KF injuries were prevalent in acute ACL-injured knees with rates of injury of 71% and 50%, respectively. ALL injuries were more frequent and more frequently severe compared to KF injuries.

## 1. Introduction

The role and relevance of the anterolateral knee structures in knee instability have been extensively explored and analyzed within the existing body of research. Numerous studies, including investigations on cadaveric specimens, have shed light on the intricate interplay of these structures in knee biomechanics. Among these, the iliotibial band (ITB) and its deep layers, notably represented by the Kaplan fibers (KF), have emerged as key components of the anterolateral complex. These structures are implicated in the control of anterior translation and internal rotation of the tibia [1,2]. Lutz et al. [3] have reinforced the role of both ALL and KF as knee stabilizers. Their research demonstrated that these structures are the primary distinct elements that become tightened during the internal rotation of the tibia, underscoring their crucial contributions to knee stability. Despite a wealth of studies and discussions on the topic, the debate still persists regarding which of these structures, be it the ALL, KF, or a combination of both, plays the central role in providing anterolateral stabilization [2,4,5]. This ongoing discourse reflects the complexities and nuances of knee biomechanics and the need for continued investigation to elucidate the precise mechanisms underlying knee stability.

More recently, a growing body of literature has brought into question the traditional understanding of the anterolateral ligament (ALL) as the predominant contributor to anterolateral knee stability, highlighting the contribution of other anatomic structures, including the deep fibers of the ITB [6]. These controversies also have an impact on anterior cruciate ligament (ACL) reconstruction techniques. Some surgeons prefer a combination of ACL and ALL reconstructions, since these are clearly defined structures with defined biomechanics; while others favor a lateral tenodesis that reinforces the deep structures of the ITB other than ALL itself [7]. However, despite the ongoing debate and the potential clinical implications of these varying approaches, the field is lacking comprehensive, high-quality studies with long-term follow-up data to definitively compare the efficacy and outcomes of these techniques in real-world clinical scenarios [8]. This knowledge gap underscores the importance of ongoing research efforts to provide the orthopedic community with evidence-based guidance and a clearer understanding of the most effective methods for restoring anterolateral knee stability.

Previous studies have consistently achieved high identification rates for both the anterolateral ligament (ALL) and the Kaplan fibers (KF) in routine magnetic resonance imaging (MRI) scans of knees, whether they have intact [9] or injured [10] ACLs. Nevertheless, despite significant advancements in imaging techniques, the evaluation of KF and ALL injuries in ACL-deficient knees has been the focus of only a limited number of investigations, yielding conflicting results and showing variable interobserver agreement. As the evaluation of anterolateral ligament (ALL) and Kaplan fibers (KF) integrity is not amenable to biomechanical tests, an accurate and reliable image assessment remains crucial for detecting abnormalities in these structures. Furthermore, data on the connection between these injuries remain notably scarce.

This study thus aimed to evaluate the rate of ALL and KF injuries in adults with acute ACL injuries using MRI. We also aimed to analyze the interobserver reproducibility in detecting these lesions and evaluate whether there is an association between ALL and KF injuries. We hypothesize both types of injuries to be common and the evaluation correlation to be satisfactory.

## 2. Materials and Methods

### 2.1. Design and Patient Selection

This study was approved by the Human Research Ethics Committee. The analysis was conducted in accordance with the ethical standards of the Declaration of Helsinki. 

We retrospectively reviewed data from 64 consecutive adult patients who had both clinical and MRI diagnoses of ACL tears, focusing on the detection of injuries to the ALL or KF. Only patients with acute injuries were included, defined as those who underwent an MRI within 2 months of the injury. Diagnoses were made by orthopedic surgeons belonging to the same team and image diagnosis was provided by musculoskeletal radiologists.

Patients who had had previous knee injuries, surgeries, or infections, as well as those with metallic material present around the knee, degenerative changes classified as Kellgren–Lawrence grade > 2, motion artifacts, or non-routine MRI protocols, were excluded from the study. 

### 2.2. Radiological Analysis 

Noncontrast MRIs were obtained using identical protocols. The knees were examined using a 1.5 T scanner (Signa Excite HD; GE Healthcare, Waukesha, WI, USA) with a dedicated knee coil (HD T/R 8-channel high-resolution knee array). The patients were placed in a supine position with their knees extended. The imaging sequences used were multiplane fast spin echo imaging optimized for anatomic detail with the following parameters: repetition time 4000–6000 ms, echo time 25–30 ms, echo train length 8–16, receiver bandwidth 32–62.5 kHz, and an acquisition matrix of 512. 3 (256 to 416). The imaging protocol for this study encompassed a comprehensive approach, incorporating T2-weighted images with fat saturation across the axial, coronal, and sagittal planes. Additionally, T2-weighted sagittal images without fat saturation and coronal T1 images without fat saturation were included in the imaging regimen. The sequences were meticulously acquired with a uniform thickness of 3.5 mm and a spacing of 0.3 mm, ensuring a detailed and high-resolution examination.

A senior musculoskeletal radiologist with 10 years of experience and a musculoskeletal fellow conducted an evaluation of 10 MRI scans of patients with ACL tears to establish a consensus regarding the qualitative MRI features of normal and injured KFs and ALLs. Both radiologists, who were independent and blinded to the clinical findings, analyzed the coronal, sagittal, and axial MRI sets of all patients. The evaluation included the assessment of ALL and KF injuries to determine interobserver agreement. Additionally, injuries to the collateral ligaments, ITB (excluding its deep fibers, which were evaluated separately), and posterior cruciate ligament (PCL) were evaluated. 

**Figure 1:**
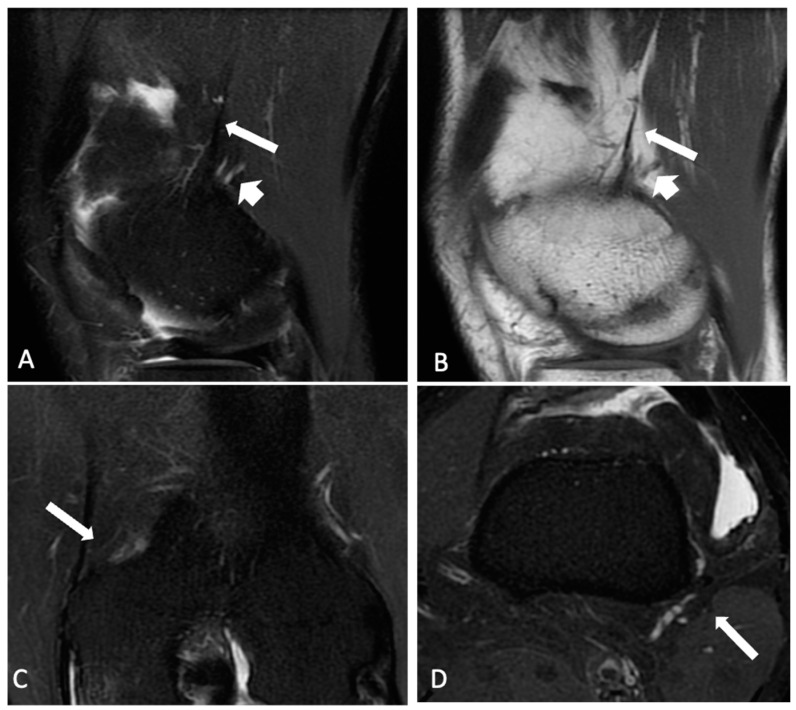
Intact Kaplan fibers. Knee MR sagittal T2FS (**A**), sagittal T1 (**B**), coronal T2FS (**C**) and axial T2FS (**D**) showing intact distal Kaplan fibers (long arrow) and geniculate arteries (short arrow).

**Figure 2:**
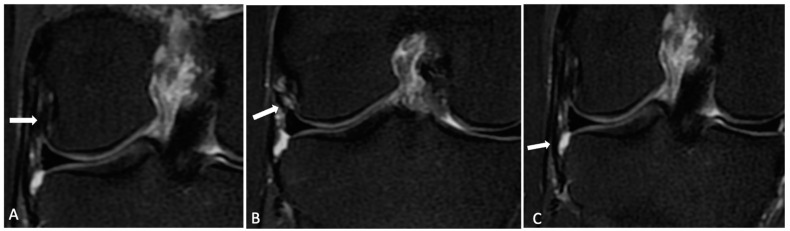
Normal anterolateral ligament. Knee MR axial T2FS images showing femoral (**A**), meniscal (**B**), and tibial (**C**) portions of the anterolateral ligament.

**Figure 3:**
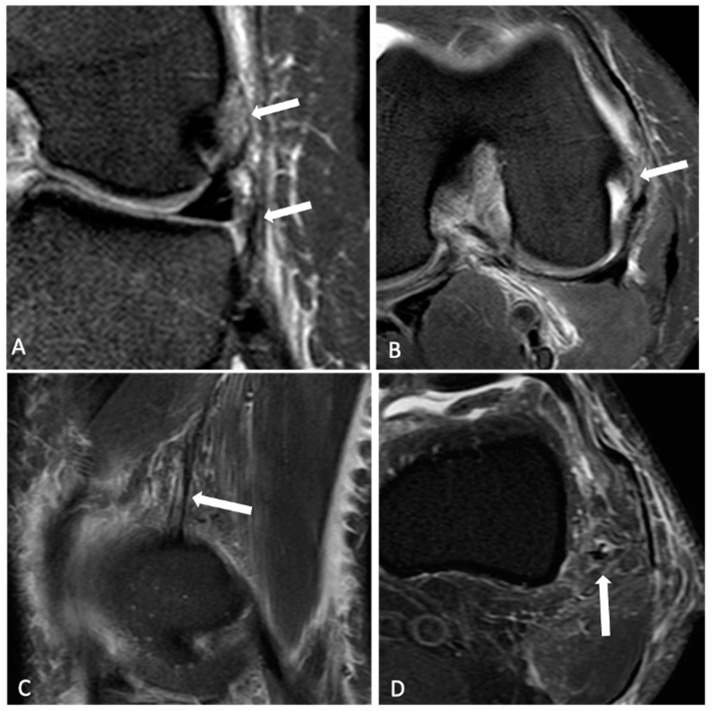
A 28-year-old patient with a partial tear of the anterolateral ligament and intact Kaplan fibers. Knee MR coronal and axial T2FS (arrows in (**A**,**B**)) showing edema and irregularity of the ALL (arrows), without discontinuity. Sagittal and axial T2FS images (arrows in (**C**,**D**)) showing intact Kaplan fibers (arrows in (**B**,**C**)).

**Figure 4:**
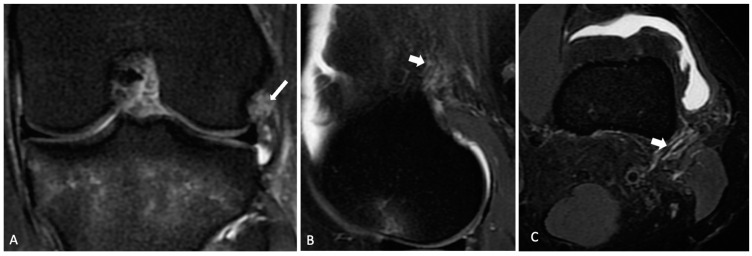
A 36-year-old patient with complete tears of the anterolateral ligament and Kaplan fibers. Knee MR coronal (**A**) image showing a complete tear with discontinuity of the meniscal portion of the anterolateral ligament (long arrow). Sagittal and axial (**B**,**C**) showing complete tear of the Kaplan fibers (short arrows).

The normal ALL was identified as a low-signal-intensity band that originated from the lateral femoral epicondyle, bifurcated above the inferior lateral geniculate vessels, and distally inserted into the body of the lateral meniscus and the lateral tibial plateau. KFs were identified with the distal fiber bundles near the branches of the superior genicular artery and the proximal bundles at the lateral ridge of the distal femur [9]. Each radiologist categorized both the ALL and KFs as intact (Figure 1 and Figure 2), partially injured (Figure 3), or completely injured (Figure 4), based on a previous publication [11]. Intact ligaments were characterized as a continuous clearly defined low-signal band. Injuries were considered in any portion of the structure. Partial injuries included patients with signal changes and edema of the fibers of the ligaments, as well as thinning or irregularity of the anterolateral structures, without a clear fiber discontinuity; complete injuries were characterized by a complete disruption of fibers or no clear continuity in any segment. 

The edema around both the ALL and KFs alone, without abnormalities of the fibers of these structures, was not considered an injury, as soft tissue edema in the lateral side of the knee is very common in ACL injuries due to the trauma, injuries of lateral structures or joint effusion and periarticular edema.

### 2.3. Statistical Analysis

The severity of each injury (ALL and KFs) was described using absolute and relative frequencies, and the association between the severities of different injuries was evaluated using chi-square tests for trends. The association between these lesions was assessed using chi-square tests or Fisher’s exact tests. Contingency tables and Kappa coefficients were calculated to assess the agreement between the examiners regarding the presence of injuries. The calculations included 95% confidence intervals (Cis). For the evaluation of the interobserver agreement only the presence or absence of the main lesions of interest, namely ALL and KF injuries, was considered. 

Data analysis was performed using IBM SPSS Statistics for Windows, version 22.0 (IBM Corp., Armonk, NY, USA), and the results were tabulated using Microsoft Excel 2013 (Microsoft Corp., Redmond, WA, USA). The significance level for the tests was set at 5%.

## 3. Results

Overall, 72 patients met the inclusion criteria for the study. After excluding 8 patients due to poor quality (*n* = 1) and advanced degenerative osteoarthritis (*n* = 7), 64 patients were ultimately included in the analysis. The mean age of the patients was 33 years (range 19–66 years), with 44 patients (66%) being male and 37 (50%) having right knee injuries. The demographics and prevalence of ligament injuries are presented in Table 1.

ALLs and KFs were adequately identified on MRI in all patients, meeting the pre-established criteria. Despite accessing all planes and sequences for both the ALL and KFs, the predominant approach for the ALL involved utilizing the coronal plane with fluid-sensitive sequences, specifically T2-weighted images with fat saturation. The oblique orientation of the ALL to the coronal plane necessitated a meticulous review of consecutive images to ensure a thorough assessment, avoiding incomplete evaluations or the impact of partial volume effects. In contrast, the observers employed all three planes for assessing the Kaplan fibers, with sagittal and axial images being particularly highlighted for their perceived reliability in accessing and characterizing KFs.

On MRI, ALL injuries were observed in 46 (71%) patients, among whom 33 (71%) had partial and 13 (28%) had complete injuries. Tibial avulsion (Segond lesion) was not detected in any of the patients. KF injuries were identified in 32 (50%) patients, with 28 (87.5%) of them having partial and 4 (12.5%) having complete injuries. Examples of intact and injured structures are shown in Figure 1, Figure 2, Figure 3 and Figure 4. Combined injuries of both ALL and KFs were found in 25 (32.4%) patients, with a *p*-value of 0.266, indicating a non-significant association.

In MRI, 28 (43%) patients had a medial collateral ligament (MCL) injury, and 24 (37.5%) had a lateral collateral ligament (LCL) injury. There were significant associations between the severity of the ALL injury and ITB, PCL, MCL, and LCL injuries (*p* < 0.05). Similarly, the severity of KF injuries was associated with injuries affecting ITB, PCL, and MCL (*p* < 0.05).

There was no significant association found between ALL, KF, and ITB injuries (*p* > 0.05) (Table 2). The agreement between the examiners regarding ALL and KF injuries ranged from moderate to substantial (Kappa between 0.55 and 0.75), with the highest agreement observed in cases of KF injuries (Kappa = 0.750)—Table 3.

## 4. Discussion

The main finding of this study was that ALLs and KFs were the most injured ligament structures associated with acute ACL injuries, with rates of injury of 71% and 50%, respectively. Although there was no statistical association between ALL and KF injuries, injuries involving both structures were prevalent (54.3%). Furthermore, ALL injuries were observed to be more frequent and more frequently severe compared to KF injuries. This study also demonstrated good interobserver reliability in detecting both ALL and KF injuries on MRI, especially for the KF.

In our study, ALL was found to be the most prevalent ligament injury in patients with acute ACL injuries (71%). Studies on the rate of ALL injuries remain controversial, with a recent systematic review reporting a wide range of rates from 10.7% to 98% in ACL injuries [12]. Balendra et al. [13] studied 100 patients with acute ACL injuries and reported ALL injuries in only 22% of them, despite a high incidence of concomitant anterolateral complex injuries. On the other hand, Barrera et al. [14] found that ALL injuries occurred in up to 77% of patients with ACL injuries and highlighted the association between ALL and other lateral knee structure injuries. Similarly, Runer et al. [10] reported higher rates of ALL injuries (58%), compared to KF injuries (21%), in a study involving 91 knees of both adult and pediatric patients, with a low rate of combined ALL and KF injuries (12%). The heterogeneity in defining injury patterns, time from injury to imaging, MRI protocols and quality, and variability in radiological interpretation contribute to the discrepancy among these studies. However, while the prevalence of ALL injuries varies widely in the literature, it appears that ALL is the anterolateral structure most commonly associated with ACL injuries [11,13]. Studies utilizing nonconventional and more detailed MRI protocols, like that of Muramatsu et al. with 3-D MRI [11] and Ferretti et al. with a bilateral protocol for assessing anterolateral injuries [15], have reported ALL injuries in around 90% of ACL injury cases.

The relevance of ALL injuries in relation to clinically proven rotatory instability, as assessed through pivot-shift tests, remains controversial. Although ALL injuries were associated with rotatory knee laxity and high-grade pivot shift in biomechanical or robotic assessments [16,17,18], in vivo evaluations have yielded conflicting results. Cavaignac et al. [19] also found in vivo evidence that ALL injuries were most often associated with high-grade pivot-shift, although statistical significance was lacking among different grades of pivot-shift. Meanwhile, Barrera et al. [14] evaluated 76 patients with ACL-deficient knee injuries and found no clinical association between ALL injuries and rotatory knee laxity. Similarly, Miyiaji et al. [20] reported no association between ALL injuries and rotatory knee laxity, both in clinical grading and quantitative evaluation. These discrepancies may be attributed to variations in the precise evaluation of the pivot-shift test or differences in the definitions of injury patterns in MRI interpretation. Despite the controversies in biomechanical studies, Sobrado et al. [21] demonstrated that patients with an associated ALL injury in MRI had worse outcomes following isolated ACL reconstruction, which may be due to the poor healing rates of the anterolateral structures [22].

Indeed, the knee anterolateral complex, particularly ALL, has been the primary focus of imaging and clinical assessment studies investigating knee instability in ACL-injured knees. However, a more recent focus has also been directed towards KFs. This shift in focus is supported by vast anatomical evidence highlighting the functional relevance of KFs, which act as a restraint to internal rotation and flexion in both ACL-intact and deficient knees, as shown by cadaveric biomechanical studies [1]. 

Previous studies have reported a broad spectrum of KF injury rates, ranging from 17% [23] to 82% [24]. In our study, we observed a KF injury rate of 50%, still lower than the reports of Khanna et al. [24] and Marom et al. [25]. Conversely, another study [23] reported a relatively low KF injury rate (17%) among 267 patients with ACL-injured knees who underwent primary ACL reconstruction. This disparity underscores the considerable variability in KF injury rates among different investigations. Marom et al. [25] suggested that the discrepancies in the reported rates of KF injuries may be attributed to differences in MRI protocols, imaging parameters, and the experience of the examiners. Although described in cadaveric dissections prior to ALL, image evaluation of KF injuries is still challenging, and only recently have studies focused on the image analysis of the KF complex in standard MRI protocols [9,26]. 

Although there is robust data pointing to the relevance of KFs in rotatory knee stability, current evidence does not suggest a correlation between KF injuries and high-grade pivot shift in acute ACL injuries within the clinical setting [27]. Devitt et al. [23] also reported no association between KF injuries and high-grade pivot shifts which he hypothesized to be related to a limited role of the KF in controlling anterolateral rotatory laxity in the context of acute ACL injuries, contrasting with biomechanical sectioning studies that have proved the KF role in anterolateral stability [2].

Only a few studies have evaluated the association between ALL and KF injuries, and most of them have reported a relatively low association between these injuries, with rates of 12.7% [10] and 19% [13], indicating independent vulnerability of these structures in the trauma setting. Our study also found no statistical significance between combined injuries of ALL and KF, which were found in 32% of patients. This lack of association may help explain the presence of high-grade pivot shifts observed in knees with ALL injuries [19] as opposed to the absence of pivot shift changes in patients with KF injuries [23]. 

The individual roles of ALL and KF injuries in knee instability are still a subject of discussion. In the clinical setting, while ALL reconstruction has been proven to be beneficial in reducing the failure rate of ACL reconstruction, as demonstrated by numerous clinical studies, systematic reviews, and meta-analyses, the role of KF reconstruction has yet to be defined [28,29]. 

Our findings reveal a significant link between the severity of ALL and KF injuries and injuries to the collateral ligaments, consistent with prior research [30]. This observed trend aligns with earlier studies conducted by Helito [31] and Van Dyck [32] who found associations between ALL and MCL injuries. Reporting associated injuries is crucial because it can act as an early warning sign for surgeons, alerting them to the potential presence of severe injuries to the ALL and knee function in cases of complete ACL injuries.

We observed a noteworthy level of consensus among the examiners when it came to the imaging evaluation of ALL and KF injuries, with the greatest level of agreement found in cases of KF injuries. This agreement between readers is probably explained by the high experience level of the readers in identifying those structures in routine knee MRI. This level of agreement contrasts with a paper from Batty [9] who found more modest interobserver reliability scores when evaluating KF, which he attributed to multifactorial factors with an emphasis on the evident learning curve in the identification of these fibers. The assessment of the ALL studies has shown a wide range of interobserver agreement levels, with some indicating moderate to substantial agreement among clinicians when evaluating the ALL ligament, while others have reported more variable assessments, with interobserver agreements ranging between 0.33 and 1.0 [12]. These findings highlight the need for standardized evaluation protocols and further research in this area, using more uniform MRI devices, MRI parameters, and examination protocols. 

This study had some limitations. First, the inherent weaknesses of a retrospective review, despite our efforts to include only patients with high-quality MRI examinations and within a specific time period between the injury being sustained and the MRI assessments. Second, we categorized the injuries into only two groups (partial and complete injuries) and included patients with edema affecting the anterolateral structures in the partial injury group. This approach may have led to an overestimation of the injury grades and could have influenced the association analysis between injuries, as edema changes can be caused by fluid or hematoma spreading from adjacent injured structures. However, this injury pattern definition is commonly used in existing studies on this subject. Furthermore, our image acquisition utilized a 1.5 T MRI scan, which could potentially limit the assessment of distinct segments of the anterolateral ligament (ALL) and Kaplan fibers (KF). However, it is worth noting that previous studies have reported a satisfactory visibility rate (82%) for all components of the ALL in routine 1.5 T MRI scans, coupled with excellent interobserver agreement [33]. Also, we deliberately opted not to include an assessment of associated meniscal injuries in our study, as the primary focus of this paper was a comprehensive evaluation of anterolateral structures, although it is noteworthy that the existing body of literature has shown a significant correlation between meniscal injuries and KF and ALL injuries [13]. However, some other authors have reported no such association in their research [30]. Additionally, MRI assessment of the anterolateral structures is challenging and subject to variability. To address this, an initial pilot study was conducted before the case analysis to establish a certain level of consistency in the image analysis, and the examinations were independently interpreted by two reviewers who were blinded to the clinical examination findings. 

Finally, this imaging-focused study serves as an initial exploration into the prevalence and correlation of anterolateral lesions. The absence of a correlation with clinical data, including the outcomes of stability tests, is acknowledged. We recognize that the correlation with physical examination and, in a subsequent phase, the association with treatment outcomes will be crucial next steps in advancing this research line. Nevertheless, we believe that this prevalence study and the correlation analysis of these lesions lay the foundation for a deeper understanding of anterolateral injuries. As we move forward, integrating clinical data and treatment outcomes will contribute significantly to the comprehensive elucidation of these lesions.

ALL and KF injuries were highly prevalent in ACL-injured knees. Such information may have clinical relevance in the treatment of rotatory unstable knees. Further studies investigating the clinical utility of MRI in detecting injuries of the knee anterolateral complex are needed to enhance our understanding of their role in knee instability and to address surgical considerations related to lateral extra-articular surgical procedures.

## Figures and Tables

**Table 1:** Demographics and prevalence of ligament injuries.

Data		
Mean age (range), years	33 (19–66)	
Gender, *n* (%)		
Male	44 (69)	
Female	20 (31)	
Injuries, *n* (%)	Yes	No
ALL	46 (72)	18
Partial	33 (72)	-
Complete	13 (28)	-
KF	32 (50)	32 (50)
Partial	24 (75)	-
Complete	8 (25)	-
ITB	2 (3.1)	62 (96.9)
PCL	9 (14)	55 (86)
MCL	28 (43.7)	36 (56.3)
LCL	24 (37.5)	40 (62.5)

ALL, anterolateral ligament; KF, Kaplan fibers; ITB, iliotibial band; ACL, anterior cruciate ligament; PCL, posterior cruciate ligament; MCL, medial collateral ligament; LCL, lateral collateral ligament.

**Table 2:** Association between ALL and KF injuries.

	ALL Injury		Total	*p*-Value
	No	Yes		
KF injury *n* (%)				0.266
No	11 (14.2)	21 (27.2)	32 (50)	
Yes	7 (9)	25 (32.4)	32 (50)	

ALL, anterolateral ligament; KF, Kaplan fibers.

**Table 3:** Inter-observer agreement in the evaluation of ALL and KF injuries.

Variable	Reader 1	Reader 2		Total	Kappa IC (95%)
		No Injury	Injury		
ALL injury, *n* (%)	No injury	16 (25)	2 (3,1)	18 (28)	0.564
	Injury	11 (17.2)	35 (55)	46 (72)	(0.364; 0.764)
	Total	27 (42.2)	37 (57.8)	64 (100)	
KF injury, *n* (%)	No injury	30 (47)	2 (3.1)	32 (50)	0.750
	Injury	6 (9)	26 (41)	32 (50)	(0.589; 0.911)
	Total	36 (56)	28 (44)	64 (100)	

ALL, anterolateral ligament; KF, Kaplan fibers; IC, intra-class coefficient.

## Data Availability

The data presented in this study are not publicly available due to privacy restrictions.
